# Factors affecting hospital readmission rates following an acute coronary syndrome: A systematic review

**DOI:** 10.1111/jocn.16122

**Published:** 2021-11-22

**Authors:** Amineh Rashidi, Lisa Whitehead, Courtney Glass

**Affiliations:** ^1^ School of Nursing and Midwifery Edith Cowan University Perth Australia

**Keywords:** acute coronary syndrome, factors, readmission, systematic review

## Abstract

**Aim:**

To synthesise quantitative evidence on factors that impact hospital readmission rates following ACS with comorbidities.

**Design:**

Systematic review and narrative synthesis.

**Data sources:**

A search of eight electronic databases, including Embase, Medline, PsycINFO, Web of Science, CINAHL, Cochrane Library, Scopus and the Joanna Briggs Institute (JBI).

**Review methods:**

The search strategy included keywords and MeSH terms to identify English language studies published between 2001 and 2020. The quality of included studies was assessed by two independent reviewers, using Joanna Briggs Institute (JBI) critical appraisal tools.

**Results:**

Twenty‐four articles were included in the review. All cause 30‐day readmission rate was most frequently reported and ranged from 4.2% to 81%. Reported factors that were associated with readmission varied across studies from socio‐demographic, behavioural factors, comorbidity factors and cardiac factors. Findings from some of the studies were limited by data source, study designs and small sample size.

**Conclusion:**

Strategies that integrate comprehensive discharge planning and individualised care planning to enhance behavioural support are related to a reduction in readmission rates. It is recommended that nurses are supported to influence discharge planning and lead the development of nurse‐led interventions to ensure discharge planning is both coordinated and person‐centred.

## 1 INTRODUCTION

Acute coronary syndrome (ACS) including unstable angina (UA) and myocardial infarction (MI) with or without ST‐segment elevation results in significant morbidity and mortality and is associated with impaired quality of life (Reed et al., 2017; World Health Organization, 2018). According to the World Health Organization, ACS is associated with 126 deaths per 100,000 people on a global scale (World Health Organization, 2018). In Australia, approximately 7300 people died due to MI in 2018, which is equivalent to 20 deaths per day (Australian Institute of Health & Welfare, 2020). ACS not only results in sudden or premature death but also in increased costs to the health care system (Hess et al., 2016; Litovchik et al., 2019; McManus et al., 2016). People with ACS are at high risk for readmission and the rates of readmission following ACS are increasing (Belitardo & Ayoub, 2015). A recent study in Brazil reported 42.6% of people who experienced ACS were readmitted between 30 and 180 days post‐discharge (Oliveira et al., 2019). Similarly, the US study of 3536 people post‐acute MI, reported a 24.5% readmission rate within one year (Dreyer et al., 2015). A study in Australia and New Zealand indicated that 25% of patients were readmitted or died within 30 days following an acute MI (Labrosciano et al., 2017). However, a Canadian study of 3411 people who experienced ACS reported the highest readmission rate at 61.7% within one year following discharge (Southern et al., 2014). Readmission to hospital is influenced by a number of factors indicating the occurrence of complications after discharge, which can impact on disease severity (Oliveira et al., 2019), and this reinforces the importance of identifying factors such as the presence of comorbidities, clinical condition and patients’ individual features both during and after hospitalisation to reduce complications that may affect readmission outcomes (Oliveira et al., 2019; Walraven et al., 2012). Readmission to hospital among ACS patients is related to increased medical costs reduced quality of life and poorer health outcomes (Budiman et al., 2016). Identifying factors that affect readmission has the potential to improve quality of life, discharge follow‐up, care coordination and reduce avoidable healthcare expenditure (Dreyer et al., 2015; Oliveira et al., 2019; Southern et al., 2014). Readmission is a multifactorial phenomenon and influenced by underlying comorbidities, patient factors, healthcare system and organisational factors (Rocca et al., 2020). To date, a study has assessed and evaluated readmission related to patients with MI (Labrosciano et al., 2017); however, readmission rates post‐ACS are not clear (Kwok et al., 2017; Nguyen et al., 2018) and further evidence on factors influencing readmission associated with ACS is minimal (Oliveira et al., 2019). Moreover, prior research on readmission rates following an ACS plus comorbidity has not been systematically reviewed and factors affecting hospital readmission are currently lacking. Therefore, improved knowledge of factors affecting readmissions is important to develop targeted strategies to reduce readmissions rates. The aim of this review was to identify factors that are associated with hospital readmission within 30 days following ACS.

## 2 AIM

To synthesise quantitative evidence on factors that impact hospital readmission rates following ACS with comorbidities.

### 2.1 Methods

A systematic review was conducted in accordance with the Joanna Briggs Institute (JBI) methodology for systematic reviews of quantitative evidence (Moher et al., 2010). This review follows the PRISMA (File S1) (Preferred Reporting Items for Systematic Reviews and Meta‐Analyses) statement reporting (Moher et al., 2010) and was registered with PROSPERO (*registration number will be inserted here post‐review).

#### 2.1.1 Search methods

Gaps in the literature were identified through an initial scoping search of Medical Literature Analysis and Retrieval System Online (MEDLINE) and Google Scholar. Keywords related to samples with acute coronary syndrome diagnosis (‘acute coronary syndrome’, ‘ACS’, ‘cardiovascular disease’) and terms related to 30‐day readmission rates (‘readmission*’, ‘rehospitalization*’, ‘repeat* admission*’) were used to identify further key terms. Once the full search strategy was established, concepts were combined with Boolean operators ‘AND’ and ‘OR’ (Appendix 1). Studies were identified through a systematic search of eight electronic databases (Embase, Medline, PsycINFO, Cumulative Index to Nursing and Allied Health Literature (CINAHL), Web of Science, Cochrane Library, Scopus and the Joanna Briggs Institute (JBI) in January 2020. Systematic review reference lists were hand searched for additional eligible literature.

#### 2.1.2 Study selection and inclusion criteria

All search results were imported into EndNote X9 with the aim of removing duplicates records. Two independent reviewers screened the titles and abstracts of the full‐text articles against the inclusion criteria set out in the protocol. All peer‐reviewed experimental and observational study designs were considered for inclusion. Only studies classified as primary research, published in English since 2001 in a peer‐reviewed journal were considered. This time frame was considered as preliminary searches indicated an upsurge in the discourse on treatment adherence potentially influencing 30‐day readmission rates. This review considered populations diagnosed with acute coronary syndrome or ischaemic‐related cardiovascular conditions and identified comorbidity on initial admission with a subsequent presentation to hospital for treatment within 30 days. The diagnosis of ACS includes the suspicion or confirmation of acute myocardial ischaemia or infarction. Non‐ST‐elevation myocardial infarction (NSTEMI), ST‐elevation MI (STEMI) and unstable angina are the three traditional classifications of ACS, which were considered for this review. This review focused on adults aged 18 years and over. Studies were excluded from the review if they included participants under 18 years old, had non‐ischaemic heart failure or had received open heart surgery. Animal studies and grey literature were excluded. This review assessed the readmission rate of patients occurring within 30 days of receiving care for an acute coronary syndrome episode in an acute care setting. The outcomes of interest for this review were the factors that influenced 30‐day readmission in patients that had an initial hospitalisation for acute coronary syndrome or ischaemic‐related cardiovascular condition. These factors could include, but were not limited to, education, treatment plan implementation and adherence, communication, quality of life, general health outcomes, physical well‐being, emotional wellbeing, comorbidities, stress or psychosocial well‐being as assessed through data retrieved from medical records/database review, patient questionnaires or patient interviews.

#### 2.1.3 Quality appraisal and data extraction

Methodological quality and risk of bias within included studies were independently appraised by two reviewers. JBI Critical appraisal tools were utilised to assess the quality of studies (Manual, 2014). Any disagreements that arose between reviewers were discussed and resolved by all authors. A Grading of Recommendations Assessment, Development and Evaluation (GRADE) was completed to evaluate the certainty of the evidence by assessing consistency of results between studies, directness and precision of findings, study limitations and probability of publication bias. The overall quality of evidence was categorised as high, moderate, low or very low. Two researchers completed the GRADE assessments independently. Any discrepancies between reviewers were discussed by the research team.

Data were extracted from included studies using the JBI standardised data extraction tools by study design (Piper, 2019). Data extracted included country, study design, type of patient (referring to reason for initial admission), sample size, sample demographics (age and sex), intervention details, data source or measurement and comorbidity or outcome of interest (referring to the factors that could impact on readmission rates), as well as key findings. Queries on data were followed up with the corresponding authors of each study. One author was contacted to gain clarification on the data, as conflicting sample characteristics were reported; however, no return correspondence was received.

#### 2.1.4 Data synthesis

Key findings have been narratively synthesised to demonstrate key barriers and facilitators for 30 days readmission rates. Meta‐analysis was not completed due to heterogeneity in data collection time points, source of data, study design and collection methods. Therefore, a narrative synthesis was conducted according to the Synthesis Without Meta‐analysis (SWiM) guidelines (Campbell et al., 2020). The review was approved by the Human Research Ethics Committee of University (2021‐02514).

## 3 RESULTS

### 3.1 Study inclusion

A PRISMA flow diagram outlining the selection of eligible studies is presented in Appendix 2. Initially, 13515 articles were retrieved from databases and manual searching. After the removal of duplicates, 9132 papers remained. These titles were screened by two independent reviewers. A total of 818 abstracts were reviewed for suitability. Full texts of 321 articles were assessed for eligibility by two independent reviewers, with 297 excluded for not meeting inclusion criteria. Reasons for exclusion included the primary diagnosis on admission (*n* = 104), patients who underwent a coronary artery bypass grafting procedure during the admission (*n* = 38), readmission outside of the 30‐day period (*n* = 62), comorbidities not reported (*n* = 80), grey literature (*n* = 7), reviews (*n* = 4), non‐adult sample (*n* = 1) and paper not published in English (*n* = 1). An overall GRADE quality rating of low was assigned to findings in relation to factors that influence 30 days readmission rates, mostly due to the nature of the study design being observational.

### 3.2 Methodological quality of included studies

A summary of the quality assessment of the included studies is presented in Appendix 3. The 24 included studies were critically appraised by two independent reviewers using the JBI checklist. Of the 22 cohort studies (maximum quality score 11), 15 studies were assigned a score of 9 (Borzecki et al., 2016; Cheung et al., 2018; Dharmarajan et al., 2013; Dodson et al., 2019; Hess et al., 2016; Khera et al., 2017; Li et al., 2019; Litovchik et al., 2019; Mahmoud & Elgendy, 2018; McHugh & Ma, 2013; McManus et al., 2016; Meadows et al., 2012; Przybysz‐Zdunek et al., 2012; Southern et al., 2014; Zabawa et al., 2018), five studies scored 11 (Dreyer et al., 2015; Kociol et al., 2012; Rodriguez et al., 2011; Tripathi et al., 2017, 2019) and the remaining studies scored 10 (Nuti et al., 2016) and 6 (Atti et al., 2019), respectively. The RCT studies scored 10 (maximum quality score 13) (Cajanding, 2017). The quasi‐experimental study scored 8/9 (Gasbarro et al., 2015).

### 3.3 Characteristics of included studies

Appendix 4 presents an overview of the study characteristics. All included studies (*n* = 24) were published in English between 2001 and 2020. This review included five prospective cohort studies (Dodson et al., 2019; Hess et al., 2016; Li et al., 2019; Litovchik et al., 2019; McManus et al., 2016), 17 retrospective cohort studies (Atti et al., 2019; Borzecki et al., 2016; Cheung et al., 2018; Dharmarajan et al., 2013; Dreyer et al., 2015; Khera et al., 2017; Kociol et al., 2012; Mahmoud & Elgendy, 2018; McHugh & Ma, 2013; Meadows et al., 2012; Nuti et al., 2016; Przybysz‐Zdunek et al., 2012; Rodriguez et al., 2011; Southern et al., 2014; Tripathi et al., 2017, 2019; Zabawa et al., 2018), one RCT (Cajanding, 2017) and one quasi‐experimental (Gasbarro et al., 2015). The sample size of studies ranged from 50 (Gasbarro et al., 2015) to 212,171 (Khera et al., 2017). The majority of studies were conducted in the USA (Atti et al., 2019; Borzecki et al., 2016; Cheung et al., 2018; Dharmarajan et al., 2013; Dodson et al., 2019; Dreyer et al., 2015; Gasbarro et al., 2015; Hess et al., 2016; Khera et al., 2017; Kociol et al., 2012; Mahmoud & Elgendy, 2018; McHugh & Ma, 2013; McManus et al., 2016; Meadows et al., 2012; Nuti et al., 2016; Tripathi et al., 2017, 2019), two studies in Canada (Kociol et al., 2012; Southern et al., 2014) and one in France (Zabawa et al., 2018), Spain (Rodriguez et al., 2011), Poland (Przybysz‐Zdunek et al., 2012), Israel (Litovchik et al., 2019), Philippines (Cajanding, 2017) and China (Li et al., 2019). Nine studies were conducted in a hospital setting (Cajanding, 2017; Dodson et al., 2019; Gasbarro et al., 2015; Hess et al., 2016; Kociol et al., 2012; Li et al., 2019; Litovchik et al., 2019; McManus et al., 2016; Przybysz‐Zdunek et al., 2012), seven studies extracted data from hospital databases, including readmission, admission and discharge data (Atti et al., 2019; Borzecki et al., 2016; Cheung et al., 2018; McHugh & Ma, 2013; Meadows et al., 2012; Southern et al., 2014; Zabawa et al., 2018), five studies used national readmission databases (Dreyer et al., 2015; Khera et al., 2017; Mahmoud & Elgendy, 2018; Tripathi et al., 2017, 2019) and three studies used Medicare service claims data (Dharmarajan et al., 2013; Nuti et al., 2016; Rodriguez et al., 2011).

### 3.4 Review findings

#### 3.4.1 Timing of readmission

There were notable differences in the weighted median and mean time to readmission across the studies. Thirteen studies did not report either median or mean time to readmission (Borzecki et al., 2016; Cajanding, 2017; Gasbarro et al., 2015; Hess et al., 2016; Khera et al., 2017; Kociol et al., 2012; Litovchik et al., 2019; McHugh & Ma, 2013; McManus et al., 2016; Meadows et al., 2012; Nuti et al., 2016; Przybysz‐Zdunek et al., 2012; Rodriguez et al., 2011). Studies reported the median time to readmission of 2 days (Dreyer et al., 2015), 5 days (Li et al., 2019), 9 days (Tripathi et al., 2019), 10 days (Cheung et al., 2018; Dharmarajan et al., 2013; Dodson et al., 2019; Tripathi et al., 2017) and 11 days (Atti et al., 2019; Mahmoud & Elgendy, 2018). One study reported the median time from discharge to readmission of 0.8 months (Southern et al., 2014).

#### 3.4.2 Readmission rates

The percentage of patients experiencing readmission within 30 days ranged from 4.2% (Litovchik et al., 2019) to 81% (Borzecki et al., 2016). Less than 10% readmission was reported in three studies (6% (Li et al., 2019)‐9% (McHugh & Ma, 2013)). Two studies reported similar readmission rates at 34% (Cajanding, 2017; Southern et al., 2014). A readmission rate of 11% was reported in three studies (Gasbarro et al., 2015; Hess et al., 2016; Kociol et al., 2012). The remaining 13 studies ranged between 12% and 25% (Atti et al., 2019; Cheung et al., 2018; Dharmarajan et al., 2013; Dodson et al., 2019; Dreyer et al., 2015; Khera et al., 2017; Mahmoud & Elgendy, 2018; McManus et al., 2016; Nuti et al., 2016; Przybysz‐Zdunek et al., 2012; Rodriguez et al., 2011; Tripathi et al., 2017; Zabawa et al., 2018).

#### 3.4.3 Factors affecting hospital readmission

Studies focused on identifying factors that affected readmission within 30 days among ACS patients and across the review studies, a variety factors related to higher hospital readmission rates within a 30‐day period. The factors have been presented in four categories: socio‐demographic factors, behavioural factors, comorbidities and cardiac disease.

#### 3.4.4 Socio‐demographic factors

Eight studies demonstrated a link between female gender and higher likelihood of readmission within 30 days (Atti et al., 2019; Dreyer et al., 2015; Hess et al., 2016; Khera et al., 2017; Mahmoud & Elgendy, 2018; Przybysz‐Zdunek et al., 2012; Tripathi et al., 2017, 2019). However, one study reported that gender was not significantly associated with readmission within 30 days (Li et al., 2019). Age was strongly associated with readmission within 30 days, with increased age associated with increased rates of readmission (Dharmarajan et al., 2013; Dodson et al., 2019; Hess et al., 2016; Khera et al., 2017; McManus et al., 2016; Przybysz‐Zdunek et al., 2012; Tripathi et al., 2019). However, three studies (Dreyer et al., 2015; Khera et al., 2017; Southern et al., 2014) reported that older age was associated with a lower risk of readmission. One study (Li et al., 2019) demonstrated that there was no association between age and readmission. Two studies suggested that lower household income was associated with high readmission rates (Cheung et al., 2018; Khera et al., 2017). Two studies (Li et al., 2019; Tripathi et al., 2017) did not report an association between household income and rates of readmission. The association between race and readmission was examined in two studies that reported non‐white patients (Hess et al., 2016) and Hispanic patients (Rodriguez et al., 2011) were more likely to be readmitted within 30 days.

#### 3.4.5 Behavioural factors

Four studies (Cheung et al., 2018; Dodson et al., 2019; Hess et al., 2016; Mahmoud & Elgendy, 2018) identified an association between smoking and readmission. Cigarette smoking was significantly associated with a higher likelihood of readmission, irrespective of whether the patient was a current smoker (Cheung et al., 2018; Mahmoud & Elgendy, 2018). One study indicated an association between the status, current smoker and readmission within 30 days (Dodson et al., 2019). However, another study identified that cigarette smoking was linked with lower rates of unplanned readmission within 30 days (Hess et al., 2016).

#### 3.4.6 Comorbidity factors

All 24 studies reported the prevalence of comorbidities including diabetes, hypertension, pulmonary disease, renal disease and anaemia. These studies provided evidence of a relationship between different comorbidities and readmission with 30 days. Seven studies reported an association between the comorbid diagnosis of diabetes and increased rates of readmission (Atti et al., 2019; Hess et al., 2016; Kociol et al., 2012; Mahmoud & Elgendy, 2018; Przybysz‐Zdunek et al., 2012; Southern et al., 2014; Tripathi et al., 2019). Likewise, three studies (Hess et al., 2016; Kociol et al., 2012; Przybysz‐Zdunek et al., 2012) reported patients with hypertension were more likely to be readmitted within 30 days. Eight studies found that pulmonary disease or complications significantly predicted readmission within 30 days (Atti et al., 2019; Cheung et al., 2018; Dreyer et al., 2015; Kociol et al., 2012; Mahmoud & Elgendy, 2018; Southern et al., 2014; Tripathi et al., 2017, 2019). Four studies reported that patients who suffered from anaemia had a higher probability of readmission (Atti et al., 2019; Cheung et al., 2018; Mahmoud & Elgendy, 2018; Tripathi et al., 2019). Another nine studies reported the relationship between renal disease and readmission within 30 days (Atti et al., 2019; Cheung et al., 2018; Hess et al., 2016; Mahmoud & Elgendy, 2018; McManus et al., 2016; Przybysz‐Zdunek et al., 2012; Southern et al., 2014; Tripathi et al., 2017, 2019; Zabawa et al., 2018).

#### 3.4.7 Cardiac disease factors

Of the 24 studies reviewed, five studies did not report cardiac factors as the principal diagnosis for hospital readmission (Borzecki et al., 2016; Gasbarro et al., 2015; Khera et al., 2017; Nuti et al., 2016; Zabawa et al., 2018). However, a recurrent ischaemic event, including re‐infarction and unstable angina, was consistently reported as the leading cause of readmission in 12 studies (Cajanding, 2017; Dharmarajan et al., 2013; Dreyer et al., 2015; Hess et al., 2016; Kociol et al., 2012; Li et al., 2019; Litovchik et al., 2019; McManus et al., 2016; Przybysz‐Zdunek et al., 2012; Rodriguez et al., 2011; Tripathi et al., 2017, 2019). Heart failure was also a common cause of hospital readmission in four studies (Atti et al., 2019; McHugh & Ma, 2013; Tripathi et al., 2017, 2019). In three studies, the principal diagnosis for hospital readmission among people with ACS included percutaneous coronary intervention (PCI) (Meadows et al., 2012; Przybysz‐Zdunek et al., 2012; Southern et al., 2014). A variety of cardiac conditions including arrhythmia (Cheung et al., 2018; Dodson et al., 2019; Mahmoud & Elgendy, 2018; McHugh & Ma, 2013), followed by atrial fibrillation (Mahmoud & Elgendy, 2018; Przybysz‐Zdunek et al., 2012) and congestive heart failure (Cheung et al., 2018; Dodson et al., 2019; Przybysz‐Zdunek et al., 2012) were reported to be associated with readmission.

## 4 DISCUSSION

To the best of our knowledge, this is the first review to assess the prevalence of readmission within 30 days among people with ACS and comorbidity. This review was based on 24 studies that met the inclusion criteria. The findings suggest that hospital readmission within 30 days varied among persons who had experienced ACS and many factors contributed to an increased risk of readmission. The most reported factors associated with an increased risk of readmission were gender, age, ethnicity, smoking status, recurrent ischaemic events, arrhythmia, heart failure, renal disease, anaemia, diabetes, hypertension and pulmonary complications. Of interest, comorbidities were often reported as related to readmission, however, cardiac disease factors were most often reported as the principal diagnosis for hospital readmission. Further clarity on the reason for readmission, for example, recurrent MI, acute kidney injury, exacerbation of heart failure or pneumonia versus the factors related to readmission, for example history of renal disease, existing lung disease and diabetes is needed within the literature to make this distinction clearer.

Hospital readmission in people with ACS is increasingly recognised as an important healthcare issue. Overall, readmission rates ranged from 4.2% in Israel (Litovchik et al., 2019) to 81% in the United States of America (Borzecki et al., 2016). Much of the variability in the readmission rate were related to two issues. Firstly, the variability in sources of data, data collection time points and study design. Secondly, no study reported the number of people readmitted against the number who survived. The findings reported do not account for variation across healthcare systems and health across societies on readmission rates. The 30‐day period post‐discharge is a crucial interval when people are most vulnerable (Dharmarajan et al., 2013; Khera et al., 2020). Reducing readmission during this intense period of recovery and adjustment requires hospital intervention that shares characteristics of patient‐centred care involving the interdisciplinary team and better discharge planning practices.

This review also indicates that readmission varied by gender. Females had a higher likelihood of readmission within 30 days compared to males. This may be because they have home/family roles, including providing care for family members, undertake more domestic work and additional caregiving responsibilities.

The influence of these factors could add to their vulnerability for readmission and can act as a potential explanation for the gender differences in readmission. This finding links with previous research in which females had worse physical and mental health outcomes after MI (Cenko et al., 2018; Shih et al., 2019). Thirty days post‐index hospitalisation, females were more likely to be readmitted than males (38.3% versus 29.6%; *p* < .001) (Wasfy et al., 2013). This disparity has been related to a number of potential factors including actions taken or omitted during the initial hospital stay, a consequence of incomplete treatment or coordination of post‐discharge care (Kwok et al., 2018) and may include the roles and responsibilities of women in providing care for children and family that impacts on recovery and makes women more susceptible to readmission (Rose et al., 1996). Further studies need to be conducted to determine why women are more likely to be readmitted than men (Dreyer et al., 2015). Our findings also suggested racial disparities in readmission rates. Hispanic and non‐white groups have a higher risk of readmission, compared with non‐Hispanic and white patients in Spain and USA, respectively (Correa‐de‐Araujo et al., 2006; Joynt et al., 2011). One study suggested that higher readmission rates among Hispanic patients may be related to lower quality care, for example Hispanics were less likely to be offered an assessment of left ventricular function, (Correa‐de‐Araujo et al., 2006; Joynt et al., 2011). This finding is echoed in a further study (Joynt et al., 2011) that highlighted disparities in readmission rates between black and white patients in the USA and related to race itself as well as to the site where care was provided.

Prominent non‐cardiac factors that influenced readmission rates were pulmonary causes or complications, hypertension, diabetes and renal disease, followed by cardiac factors, including recurrent ischaemic event, PCI, arrhythmia and heart failure. Having the diagnosis of ACS and the presence of comorbidities places people at high risk for hospital readmission (Condon & Mcdonnell, 2012; Gandjour et al., 2012). Reported comorbid conditions varied by studies, some studies considered the number of chronic conditions or the Charlson comorbidity score to assess comorbidity. A similar study also indicated that patients with acute myocardial infarction (AMI) and diabetes were twice as likely to be readmitted than those without diabetes (Tavani et al., 2002). Those who experienced an AMI and were diagnosed with renal disease are high risk of readmission within 30 days (Dunlay et al., 2012). Indeed, previous studies indicated that 8% of people were readmitted within 30 days after PCI (Yost et al., 2013). However, a recent review suggested that there is still ongoing debate on whether PCI can decrease 30‐day readmission rates (Wang et al., 2019). Studies indicated that 58% of recurrent coronary readmissions were due to unstable angina (Yudi et al., 2019). This review summarised the factors that influenced readmission within 30 days following ACS. It highlights the need for early follow‐up with attention to the predictors of readmission, and interventions to reduce readmissions within 30 days for this population.

## 5 STRENGTHS AND LIMITATIONS

The strength of this review was the implementation of a thorough search strategy across eight databases. The JBI manual provided a comprehensive guide to conducting this systematic review. However, this review has limitations. First, due to heterogeneity among included studies, a meta‐analysis was not possible; therefore, a narrative review of the existing evidence was provided. Secondly, the inclusion of English only studies could result in relevant studies being missed. Thirdly, heterogeneity among sampling made some comparisons challenging. Apart from three studies, readmission rates based on living conditions and marital status was not reported. Further investigation is needed to examine if these factors play a role in readmission rates, and why marital status. Fourthly, most of reviewed studies were retrospective and prospective designs, and limited RCT and experimental studies have been conducted to demonstrate the cause and effect of relationships between factors. Lastly, studies have used information from databases, administrative data and clinical data making it difficult to generalise some of the findings. Further research is needed to examine readmission following medical index admissions, and to develop and evaluate the effectiveness of nurse‐led interventions to reduce readmission among this population. Such interventions need to be integrated with cardiology services to target conditions with the highest rates of readmission in order to improve patient's outcome and reduce preventable readmissions.

## 6 CONCLUSION AND IMPLICATION FOR PRACTICE

This review identified comorbidities and cardiac factors that were associated with readmission and there is clear and convincing evidence that hospital readmission is prevalent in patients with ACS. Recurrent ischaemic events, heart failure and arrythmias were the principal cardiac factors related to readmission. Meanwhile, common comorbidities such as kidney disease, hypertension, diabetes and pulmonary complications were significant factors related to readmission. However, little evidence indicates the degree to which readmission may be preventable. Some factors such as smoking, with diabetes, anaemia and hypertension are modifiable factors and can be influenced by nurses. Therefore, efforts among nurses to reduce readmission could include comprehensive discharge planning, individualised care planning, behavioural support and home visits.

Interventions that address patient need, especially unmet needs have the potential to improve health outcomes and reduce readmission rates. To effectively reduce hospital readmissions in individuals with ACS, nurses can identify individuals at high risk for readmission based on the risk factors highlighted in this review. Addressing the issues related to disparities is challenging; however, the evidence has highlighted differences in the investigations undertaken, care provided and attention to discharge planning. The findings in this review highlight the key areas related to readmission and those areas that are modifiable to inform and shape the development of targeted strategies to reduce readmission rates among people post‐ACS.

## 7 RELEVANCE OF CLINICAL PRACTICE

- This review highlights that healthcare providers, particularly nurses need to provide comprehensive and individualised patient‐centred interventions to reduce readmission by identifying factors related to readmission and unmet patient needs.
- Nurses play an important role in supporting patients and providing continuity and individualisation of care. Adopting the recommended strategies may help nurses within the clinical practice setting both pre‐ and post‐hospital discharge to reduce readmission.

## CONFLICT OF INTEREST

The authors declared that there is no conflict of interest.

## Supporting information

Supplementary Material

## Data Availability

All data generated or analysed during this study are included in this published article (Appendix 4).

## APPENDIX 1

### SEARCH STRATEGY

#### Medline: (Filtered for 2001–2020 and English)

((""cardiovascular disease"" OR (MH "Cardiovascular Diseases")) OR (""coronary artery disease*"" OR (MH "Coronary Artery Disease") OR (MH "Coronary Disease")) OR (""coronary heart disease*"") OR ((MH "Acute Coronary Syndrome") OR ""acute coronary syndrome"") OR ((MH "Myocardial Infarction") OR ""myocardial infarct*"") OR ((MH "Angina, Unstable") OR ""unstable angina"") OR ((MH "Myocardial Ischemia") OR ""myocardial ischemia"") OR (""ischemic heart failure"" OR (MH "Heart Failure")) OR "CVD" OR "CAD" OR "MI" OR "CHD" OR ((MH "Heart Arrest") OR ""cardiac arrest"") OR (""heart disease"") OR (""heart attack"") OR ((MH "Heart Diseases")) OR "ACS") AND (((MH "Patient Readmission") OR ""readmission*"") OR (""readmission rate*"") OR (""readmission rates within 30 days"") OR ("repeat* admission*") OR "rehospitali#ation" OR ("re‐admission" OR "re‐hospitalization") OR ("readmission rates in hospital*")) AND ("factor" OR "reason*" OR "influence*" OR "cause*") AND ((""acute setting*"") OR "hospital*").

#### CINAHL: (Filtered for 2001–2020 and English)

((""cardiovascular disease"" OR (MH "Cardiovascular Diseases")) OR (""coronary artery disease*"" OR (MH "Coronary Artery Disease") OR (MH "Coronary Disease")) OR (""coronary heart disease*"") OR ((MH "Acute Coronary Syndrome") OR ""acute coronary syndrome"") OR ((MH "Myocardial Infarction") OR ""myocardial infarct*"") OR ((MH "Angina, Unstable") OR ""unstable angina"") OR ((MH "Myocardial Ischemia") OR ""myocardial ischemia"") OR (""ischemic heart failure"" OR (MH "Heart Failure")) OR "CVD" OR "CAD" OR "MI" OR "CHD" OR ((MH "Heart Arrest") OR ""cardiac arrest"") OR (""heart disease"") OR (""heart attack"") OR ((MH "Heart Diseases")) OR "ACS") AND (((MH "Patient Readmission") OR ""readmission*"") OR (""readmission rate*"") OR (""readmission rates within 30 days"") OR ("repeat* admission*") OR "rehospitali#ation" OR ("re‐admission" OR "re‐hospitalization") OR ("readmission rates in hospital*")) AND ("factor" OR "reason*" OR "influence*" OR "cause*") AND ((""acute setting*"") OR "hospital*").

#### PsycINFO: (Filtered for 2001–2020 and English)

((""cardiovascular Disease"" OR (MH "Cardiovascular Diseases")) OR (""coronary artery disease*"" OR (MH "Coronary Artery Disease") OR (MH "Coronary Disease")) OR (""coronary heart disease*"") OR ((MH "Acute Coronary Syndrome") OR ""acute coronary syndrome"") OR ((MH "Myocardial Infarction") OR ""myocardial infarct*"") OR ((MH "Angina, Unstable") OR ""unstable angina"") OR ((MH "Myocardial Ischemia") OR ""myocardial ischemia"") OR (""ischemic heart failure"" OR (MH "Heart Failure")) OR "CVD" OR "CAD" OR "MI" OR "CHD" OR ((MH "Heart Arrest") OR ""cardiac arrest"") OR (""heart disease"") OR (""heart attack"") OR ((MH "Heart Diseases")) OR "ACS") AND (((MH "Patient Readmission") OR ""readmission*"") OR (""readmission rate*"") OR (""readmission rates within 30 days"") OR ("repeat* admission*") OR "rehospitali#ation" OR ("re‐admission" OR "re‐hospitalization") OR ("readmission rates in hospital*")) AND ("factor" OR "reason*" OR "influence*" OR "cause*") AND ((""acute setting*"") OR "hospital*").

#### Cochrane: (Filtered for 2001–2020)

("acute coronary syndrome" OR "myocardial infarct*" OR "cardiovascular disease" OR "coronary artery disease*" OR "coronary heart disease*" OR "unstable angina" OR "myocardial ischemia" OR "ischemic heart failure" OR " CVD" OR "CAD" OR "ACS" OR "MI" OR "CHD" OR "heart attack" OR "cardiac arrest" OR "heart disease*") AND ("readmission rate*" OR "readmission rates within 30 days" OR "readmission*" OR "repeat* admission*" OR "rehospitali#ation" OR "re‐admission*" OR "readmission rate in hospital*") AND ("factor*" OR "cause*" OR "reason*" OR "influence") AND ("hospital*" OR "acute setting*").

#### WoS: (Filtered for 2001–2020 and English)

("acute coronary syndrome" OR "myocardial infarct*" OR "cardiovascular disease" OR "coronary artery disease*" OR "coronary heart disease*" OR "unstable angina" OR "myocardial ischemia" OR "ischemic heart failure" OR " CVD" OR "CAD" OR "ACS" OR "MI" OR "CHD" OR "heart attack" OR "cardiac arrest" OR "heart disease*") AND ("readmission rate*" OR "readmission rates within 30 days" OR "readmission*" OR "repeat* admission*" OR "rehospitali#ation" OR "re‐admission*" OR "readmission rate in hospital*") AND ("factor*" OR "cause*" OR "reason*" OR "influence") AND ("hospital*" OR "acute setting*").

#### Embase

(‘cardiovascular disease'/exp OR ‘acute coronary syndrome'/exp OR ‘heart infarction'/exp OR ‘coronary artery disease'/exp OR ‘ischemic heart disease'/exp OR ‘coronary heart disease’ OR ‘myocardial infarction’ OR ‘unstable angina pectoris'/exp OR ‘heart muscle ischemia'/exp OR ‘ischemic heart failure'/exp OR cvd OR cad OR acs OR mi OR chd OR ‘heart attack’ OR ‘heart disease'/exp OR ‘heart arrest'/exp) AND (‘hospital readmission'/exp OR ‘readmission rate’ OR ‘readmission rate within 30 days’ OR readmission OR ‘repeated admission’ OR rehospitalisation OR hospitalisation OR ‘re admission’ OR ‘re hospitalization’ OR ‘re hospitalization’ OR ‘readmission rate in hospital’) AND (factors OR influence OR cause OR reason) AND (‘hospital'/exp OR ‘acute setting’) AND (2001:py OR 2002:py OR 2003:py OR 2004:py OR 2005:py OR 2006:py OR 2007:py OR 2008:py OR 2009:py OR 2010:py OR 2011:py OR 2012:py OR 2013:py OR 2014:py OR 2015:py OR 2016:py OR 2017:py OR 2018:py OR 2019:py OR 2020:py) AND [humans]/lim AND [english]/lim.

#### Scopus

((TITLE‐ABS‐KEY ("readmission rates in hospital")) OR (TITLE‐ABS‐KEY ("re‐admission*")) OR (TITLE‐ABS‐KEY ("rehospitalization*")) OR (TITLE‐ABS‐KEY ("repeat* admission*")) OR (TITLE‐ABS‐KEY ("readmission rate*")) OR (TITLE‐ABS‐KEY (readmission*)) OR (TITLE‐ABS‐KEY ("readmission rates within 30 days"))) AND ((TITLE‐ABS‐KEY ("influence*")) OR (TITLE‐ABS‐KEY ("reason*")) OR (TITLE‐ABS‐KEY (cause*)) OR (TITLE‐ABS‐KEY (factor*))) AND ((TITLE‐ABS‐KEY (hospital*)) OR (TITLE‐ABS‐KEY ("acute setting*"))) AND ((TITLE‐ABS‐KEY ("cardiovascular disease")) OR (TITLE‐ABS‐KEY ("coronary heart disease*")) OR (TITLE‐ABS‐KEY ("coronary artery disease*")) OR (TITLE‐ABS‐KEY ("acute coronary syndrome")) OR (TITLE‐ABS‐KEY ("myocardial infarct*")) OR (TITLE‐ABS‐KEY ("unstable angina")) OR (TITLE‐ABS‐KEY ("myocardial ischemia")) OR (TITLE‐ABS‐KEY ("ischemic heart failure")) OR (TITLE‐ABS‐KEY (cvd)) OR (TITLE‐ABS‐KEY (cad)) OR (TITLE‐ABS‐KEY (acs)) OR (TITLE‐ABS‐KEY (mi)) OR (TITLE‐ABS‐KEY (chd)) OR (TITLE‐ABS‐KEY ("heart disease*")) OR (TITLE‐ABS‐KEY ("heart attack")) OR (TITLE‐ABS‐KEY ("cardiac arrest"))) AND (LIMIT‐TO (PUBYEAR, 2020) OR LIMIT‐TO (PUBYEAR, 2019) OR LIMIT‐TO (PUBYEAR, 2018) OR LIMIT‐TO (PUBYEAR, 2017) OR LIMIT‐TO (PUBYEAR, 2016) OR LIMIT‐TO (PUBYEAR, 2015) OR LIMIT‐TO (PUBYEAR, 2014) OR LIMIT‐TO (PUBYEAR, 2013) OR LIMIT‐TO (PUBYEAR, 2012) OR LIMIT‐TO (PUBYEAR, 2011) OR LIMIT‐TO (PUBYEAR, 2010) OR LIMIT‐TO (PUBYEAR, 2009) OR LIMIT‐TO (PUBYEAR, 2008) OR LIMIT‐TO (PUBYEAR, 2007) OR LIMIT‐TO (PUBYEAR, 2006) OR LIMIT‐TO (PUBYEAR, 2005) OR LIMIT‐TO (PUBYEAR, 2004) OR LIMIT‐TO (PUBYEAR, 2003) OR LIMIT‐TO (PUBYEAR, 2002) OR LIMIT‐TO (PUBYEAR, 2001)) AND (LIMIT‐TO (LANGUAGE, "English")).

#### JBI

(“acute coronary syndrome” OR “heart disease” OR “myocardial infarction”) AND (“readmission” OR “rehospitalization”) AND (“factor*” OR “cause*” OR “reason*” OR “influence”) AND (“hospital*” OR “acute setting*”).

## APPENDIX 3

### METHODOLOGICAL QUALITY OF STUDIES

#### JBI critical appraisal checklist for cohort studies

**Table:**

Citation	Q1	Q2	Q3	Q4	Q5	Q6	Q7	Q8	Q9	Q10	Q11	Score/11
Atti et al. (2019)	Y	Y	Y	U	U	Y	Y	U	U	U	Y	6/11
Borzecki et al. (2016)	Y	Y	Y	U	U	Y	Y	Y	Y	Y	Y	9/11
Cheung et al.(2018)	Y	Y	Y	NA	NA	Y	Y	Y	Y	Y	Y	9/11
Dharmarajan et al. (2013)	Y	Y	Y	U	U	Y	Y	Y	Y	Y	Y	9/11
Dodson et al. (2019)	Y	Y	Y	U	U	Y	Y	Y	Y	Y	Y	9/11
Dreyer et al. (2015)	Y	Y	Y	Y	Y	Y	Y	Y	Y	Y	Y	11/11
Hess et al. (2016)	Y	Y	Y	N	N	Y	Y	Y	Y	Y	Y	9/11
Khera et al. (2017)	Y	Y	Y	U	U	Y	Y	Y	Y	Y	Y	9/11
Kociol et al. (2012)	Y	Y	Y	Y	Y	Y	Y	Y	Y	Y	Y	11/11
Li et al. (2019)	Y	Y	Y	U	U	Y	Y	Y	Y	Y	Y	9/11
Litovchik et al. (2019)	Y	Y	Y	U	U	Y	Y	Y	Y	Y	Y	9/11
Mahmoud & Elgendy (2018)	Y	Y	Y	NA	NA	Y	Y	Y	Y	Y	Y	9/11
McManus et al., (2016)	Y	Y	Y	NA	NA	Y	Y	Y	Y	Y	Y	9/11
McHugh & Ma (2013)	Y	Y	Y	NA	NA	Y	Y	Y	Y	Y	Y	9/11
Meadows et al. (2012)	Y	Y	Y	NA	NA	Y	Y	Y	Y	Y	Y	9/11
Nuti et al. (2016)	Y	Y	Y	Y	Y	Y	Y	NA	NA	NA	Y	10/13
Przybysz‐Zdunek et al. (2012)	Y	Y	Y	NA	NA	Y	Y	Y	Y	Y	Y	9/11
Rodriguez et al. (2011)	Y	Y	Y	Y	Y	Y	Y	Y	Y	Y	Y	11/11
Southern et al. (2014)	Y	Y	Y	NA	NA	Y	Y	Y	Y	Y	Y	9/11
Tripathi et al. (2017)	Y	Y	Y	Y	Y	Y	Y	Y	Y	Y	Y	11/11
Tripathi et al. (2019)	Y	Y	Y	Y	Y	Y	Y	Y	Y	Y	Y	11/11
Zabawa et al. (2018)	Y	Y	Y	NA	NA	Y	Y	Y	Y	Y	Y	

Abbreviation

Y, yes; N, no; U, unclear; NA; not applicable.

Questions:

1. Were the two groups similar and recruited from the same population?
2. Were the exposures measured similarly to assign people to both exposed and unexposed groups?
3. Was the exposure measured in a valid and reliable way?
4. Were confounding factors identified?
5. Were strategies to deal with confounding factors stated?
6. Were the groups/participants free of the outcome at the start of the study (orat the moment of exposure)?
7. Were the outcomes measured in a valid and reliable way?
8. Was the follow‐up time reported and sufficient to be long enough for outcomes to occur?
9. Was follow‐up complete, and if not, were the reasons to loss to follow‐up described and explored?
10. Were strategies to address incomplete follow‐up utilised?
11. Was appropriate statistical analysis used?

#### JBI Citical Appraisal Checklist for Quasi‐experimental studies

**Table:**

Citation	Q1	Q2	Q3	Q4	Q5	Q6	Q7	Q8	Q9	Score /9
Gasbarro et al. (2015)	Y	Y	U	Y	Y	Y	Y	Y	Y	8/9

Abbreviation

Y, yes; N, no; U, unclear; NA; not applicable.

Questions:

1. Is it clear in the study what is the ‘cause’ and what is the ‘effect’ (i.e. there is no confusion about which variable comes first)?
2. Were the participants included in any comparisons similar?
3. Were the participants included in any comparisons receiving similar treatment/care, other than the exposure or intervention of interest?
4. Was there a control group?
5. Were there multiple measurements of the outcome both pre and post the intervention/exposure?
6. Was follow‐up complete and if not, were differences between groups in terms of their follow‐up adequately described and analysed?
7. Were the outcomes of participants included in any comparisons measured in the same way?
8. Were outcomes measured in a reliable way?
9. Was appropriate statistical analysis used?

#### JBI Critical Appraisal Checklist for Randomised Controlled Trials studies

**Table:**

Citation	Q1	Q2	Q3	Q4	Q5	Q6	Q7	Q8	Q9	Q10	Q11	Q12	Q13	Score /13
Cajanding(2017)	Y	Y	Y	N	N	N	Y	Y	Y	Y	Y	Y	Y	10/13

Abbreviation

Y, yes; N, no; U, unclear; NA; not applicable.

1. Was true randomisation used for assignment of participants to treatment groups?
2. Was allocation to treatment groups concealed?
3. Were treatment groups similar at the baseline?
4. Were participants blind to treatment assignment?
5. Were outcomes assessors blind to treatment assignment?
6. Were outcomes assessors blind to treatment assignment?
7. Were treatment groups treated identically other than the intervention of interest?
8. Was follow‐up complete and if not, were differences between groups in terms of their follow‐up adequately described and analysed?
9. Were participants analysed in the groups to which they were randomised?
10. Were outcomes measured in the same way for treatment groups?
11. Was Were outcomes measured in a reliable way?
12. Was appropriate statistical analysis used?

## APPENDIX 4

### CHARACTERISTICS OF INCLUDED STUDIES FOR METHODOLOGICAL REVIEW

**Table:**

Study	Country	Study design	Participants characteristics & sample size	Intervention description	Outcome measured	Data source & objectives	Comorbidity^a^/ Outcome(s) of Interest	Complications during initial admission
Atti et al. (2019)	USA	Retrospective cohort	AMI and PCI with cariogenic shock. Sample: 46435 Age: 52.6% ≤65. Sex (%Female): 33.7%	NA	Primary outcome was 30‐day readmission rate. Secondary outcomes were predictors of readmission and cost of care associated with the index hospitalisation.	Nationwide Readmission Database	Hypertension, Diabetes, HF, prior MI, prior PCI, prior CABG, prior stroke, AF, Ventricular tachycardia/fibrillation, Peripheral vascular disease, Anaemia, Coagulopathy, Chronic pulmonary disease, Chronic kidney disease, Neurological disorders/paralysis	Major bleeding, vascular complications, stroke/TIA, respiratory complications, sepsis, AKI requiring dialysis
Borzecki et al. (2016)	USA	Retrospective cohort	Veteran's affair patients with AMI. Sample: 4986 Age: Not fully abstracted data = 69.8, fully abstracted data = 70.8 Sex (%Female): Not fully abstracted data = 1.6, fully abstracted data = 3.0	NA	processes of care between Potentially Preventable Readmissions software‐flagged and nonflagged cases.	2006 to 2010 national VA administrative data. To assess whether the PPR algorithm identifies preventable readmissions, we compared processes of care between PPR software‐flagged and nonflagged cases	Heart failure, valvular disease, peripheral vascular disease, diabetes, hypertension, chronic pulmonary disease, renal failure, metastatic cancer, primary cancer, depression, alcohol abuse, CAD, hypertension, hyperlipidaemia, smoking, chronic kidney disease	
Cajanding (2017)	UK	RCT	AMI Sample: 143 Age: 14.7% ≤40 28.7% 41–50 35.7% 51–60 16.7% 61–70 4.9% ≥71 Sex (%Female): 37.1	Intervention group received usual care plus the intervention of the structured discharge planning program. This comprises of a series of personalised lecture discussions, feedback, collaborative problem solving, goal setting and action planning that was conducted. 3 consecutive daily sessions lasting between 30 and 45 minutes.	The effectiveness of a nurse‐led structured discharge planning program on perceived functional status, cardiac self‐efficacy, patient satisfaction, and unexpected hospital revisits among Filipino patients with AMI.	Minnesota Living with Heart Failure Questionnaire, Cardiac self‐efficacy questionnaire, short‐form patient satisfaction questionnaire. To determine the effectiveness of a nurse‐led structured discharge planning program on perceived functional status, cardiac self‐efficacy, patient satisfaction, and unexpected hospital revisits among Filipino patients with AMI.	Angina, hypertension, diabetes, stroke, asthma or COPD, peripheral vascular disease	
Cheung et al. (2018)	USA	Retrospective cohort	Catheter ablation of myocardial infarction‐associated ventricular tachycardia. Sample: 4000 Age: µ = 66.3 Sex (%Female): 11.3	NA	In‐hospital outcomes, costs, and 30‐day readmissions after catheter ablation of myocardial infarction–associated VT.	Nationwide readmissions database. To examine in‐hospital outcomes, costs, and 30‐day readmissions after catheter ablation of myocardial infarction–associated VT.	HF, Prior PPM/ICD, PCI, CABG, hypertension, diabetes, hyperlipidaemia, obesity, stroke, valvular heart disease, peripheral vascular disease, pulmonary hypertension, chronic lung disease, renal disease, cancer, anaemia, coagulopathy, smoking, alcohol abuse	
Dharmarajan et al. (2013)	USA	Retrospective cohort	AMI. Sample: 108992 Age: 65–74 = 28.6% 75–84 = 40.9% 85 += 30.5% Sex (%Female): 53.6%	NA	(1) the percentage of 30‐day readmissions occurring on each day (0–30) after discharge; (2) the most common readmission diagnoses occurring during cumulative time periods (days 0–3, 0–7, 0–15, and 0–30) and consecutive time periods (days 0–3, 4–7, 8–15, and 16–30) after hospitalisation; (3) median time to readmission for common readmission diagnoses; and (4) the relationship between patient demographic characteristics and readmission diagnoses and timing.	2007–2009 Medicare fee‐for‐service claims data. To examine readmission diagnoses and timing among Medicare beneficiaries readmitted within 30 days after hospitalisation for heart failure, acute myocardial infarction, or pneumonia.	HF, AMI, Renal disorder, pneumonia, arrythmias and conduction disorders	
Dodson et al. (2019)	USA	Prospective cohort	AMI. Sample: 3006 Age: µ = 81.5 Sex (%Female):44.4	NA	The outcome was all‐cause readmission at 30 days.	Patient Health Questionnaire to assess depression, telephone interview for cognitive status for cognitive ability, Seattle Angina questionnaire, SF−12 health status measures, functional assessment and review of medical record to assess presence of comorbid disease. To develop and validate an AMI readmission risk model for older patients that considered functional impairments and was suitable for use before hospital discharge.	Hypertension, Dyslipidaemia, arrythmia, heart failure, prior MI, prior revascularisation procedure, peripheral artery disease, valvular disease, stroke, Diabetes, COPD, smoking, disability, cognitive impairment, depression	
Dreyer et al. (2015)	USA	Retrospective cohort	AMI. Sample: 4775 Age: 18–65 Sex (%Female): 36.4	NA	Sex differences in the rate, timing, and principal diagnoses of 30‐day readmissions, including the independent effect of sex following adjustment for confounders.	Healthcare Cost and Utilisation Project State Inpatient Dataset. Examined sex differences in the rate, timing, and principal diagnoses of 30‐day readmissions, including the independent effect of sex following adjustment for confounders	HF, AMI, UA and other acute ischaemic HD, Chronic angina and CAD, valvular heart disease, congenital/hypertensive disease, arrythmias and conduction disorders, syncope, stroke/TIA, pulmonary embolism, peripheral vascular disease, pneumothorax, cardio‐respiratory failure, COPD, pneumonia, sepsis, UTI, cellulitis, CD infection, renal failure, anaemia, gastrointestinal haemorrhage, diabetes, lung fibrosis/other conditions, hip fracture, other lung disorders, cancer (all stratified by men and women)	
Gasbarro et al. (2015)	USA	Quasi‐experimental	AMI. Sample: 50 Age: µ = 62.0 Sex (%Female): 34.0	Clinical pharmacist intervention encompassing education (using the teach back method) and counselling addressing names of medications, indications, dosages, adverse effects, medication adherence, encouragement of exercise, alcohol and smoking advice and cost concerns. This education occurred once prior to discharge, with a follow‐up phone call within 48 hours of discharge.	The primary outcome was the all‐cause 30‐day readmission rate for AMI patients	Medical chart reviews and patient interviews. To evaluate the overall effect of clinical pharmacist interventions on preventing hospital readmissions and improving the health of patients with AMI. Secondary objectives include identifying trends in the demographic characteristics of AMI patients, identifying potential barriers to adherence, and assessing the average time spent by a pharmacist counselling AMI patients	Obesity associated with the following factors: polypharmacy/medication adherence, passed teach back counselling, cardiac readmissions including stent thrombosis, atherosclerosis and diastolic heart failure.	
Hess et al. (2016)	USA	Prospective cohort study	AMI with PCI treatment. Sample: 12312, Not readmitted = 10,986, Readmitted = 1326 Age: Not Readmitted Mdn = 59.0, Readmitted Mdn = 61.5 Sex (%Female): Not Readmitted Mdn = 27.6 Readmitted Mdn = 31.8	NA	Our primary outcome was unplanned rehospitalisations (inpatient or observation status) within 30 days after discharge.	Hospital medical and billing data as well as participant phone call confirmation of readmission. To examine rates of unplanned rehospitalisations among patients of all ages within 30 days of the index hospitalisation for acute MI, to assess hospital‐level variation in 30‐day unplanned rehospitalisations, and to identify Factors associated with 30‐day unplanned Rehospitalisations.	BMI, smoking, hypertension, dyslipidaemia, cerebrovascular disease, stroke/TIA, AF, Peripheral artery disease, chronic lung disease, diabetes, previous MI or PCI or CABG, previous HF, GI bleeding, dialysis	MI, HF, cardiogenic shock
Khera et al. (2017)	USA	Retrospective cohort	AMI. Sample: 212171 Age: µ = 66.9 Sex (%Female): 37.9	NA	Monthly risk‐adjusted rates of in‐hospital and 30‐day Post‐discharge mortality.	National readmission database. To evaluate whether the announcement or the implementation of HRRP was associated with an increase in either in‐hospital or 30‐day post‐discharge mortality following hospitalisation for AMI, HF, or pneumonia.	Age, heart failure, chronic atherosclerosis, cardiac arrythmia, valvular disease, stroke/TIA, cerebrovascular disease, paralysis, peripheral vascular disease, diabetes, AKI, end‐stage renal disease/haemodialysis, chronic kidney disease, COPD, pneumonia, asthma, fluid/electrolyte disorder, sepsis, solid malignancy, leukaemia/metastatic malignancy, anaemia, chronic skin ulcer, delirium/dementia, malnutrition, previous MI or PCI or CABG, chest pain, sepsis, dysrhythmias, renal failure, myocarditis, hypertension, gastrointestinal haemorrhage, cerebrovascular disease	Complications of devices/implant, complication from surgery or medical care
Kociol et al. (2012)	USA/Canada	Retrospective cohort	STEMI with PCI. Sample: 5745 Age: µ = 61 Sex (%Female):22.6	NA	Predictors of 30‐day post‐discharge all‐cause and nonelective readmissions.	Hospital data and case report forms. To determine international variation in and predictors of 30‐day readmission rates after STEMI and country‐level care patterns.	hypertension, CAD, COPD, AF, multivessel disease, chronic inflammatory condition, recurrent ischaemia, prior CABG, HF, chronic liver disease, dialysis, diabetes, smoking	
Li et al. (2019)	China	Prospective cohort study	AMI. Sample: 3387 Age: Mdn = 61 Sex (%Female): 23.1	NA	Our primary outcome was the 30‐day unplanned all‐cause readmission, defined as the first unplanned rehospitalisation to an acute care hospital within 30 days from the date of discharge. Death events by death certificate or record in death cause registration system.	Chart abstraction, patient interviews and central laboratory analysis. To calculate rates of unplanned readmissions after hospitalisation for AMI, characterised readmission timing and diagnoses, and identified predictors of both unplanned all cause and unplanned cardiovascular readmissions	MI, single‐vessel disease, multiple vessel disease, prior MI or PCI, hypertension, diabetes, dyslipidaemia, smoking, prior stroke, prior chronic renal dysfunction and prior HF	AF, recurrent angina, recurrent AMI, tachycardia, HF, infection, stroke, bleeding
Litovchik et al. (2019)	Israel	Prospective cohort study	ACS. Sample: 13010 Age: µ = 63.0 Sex (%Female): 23.0	NA	incidence and outcomes of patients readmitted after an acute coronary syndrome	Hospital records, follow‐up visits and a telephone call at 30 days. To explore the prognosis of readmitted patients, and analysed national trends in readmission rates following ACS over the past decade.	Smoking, family hx of CAD, dyslipidaemia, hypertension, diabetes, chronic renal failure, COPD, peripheral vascular disease, stroke/TIA, past MI, congestive HF, unstable angina	
Mahmoud (2018)	USA	Retrospective cohort	AMI with cardiogenic shock. Sample: 39807 Age: µ = 66.5 Sex (%Female): 33.2	NA	The primary outcome of interest was 30‐day all‐cause readmission.	National readmission database. To compare 30‐day readmissions in women versus men initially admitted with AMI complicated with cardiogenic shock.	prior MI, Prior PCI, prior CABG, stroke, CAD, smoking, dyslipidaemia, AIDS, anaemia, rheumatologic disease, chronic blood loss, CHF, COPD, coagulopathy, depression, diabetes, drug abuse, hypertension, hypothyroidism, chronic liver disease, lymphoma, fluid and electrolyte disturbance, metastatic cancer, neurological disorders, obesity, paralysis, peripheral vascular disease, psychosis pulmonary circulatory disorder, chronic renal failure, peptic ulcer disease, valvular heart disease, weight loss	Acute renal failure, pneumonia, gastrointestinal bleeding, intracranial haemorrhage, stroke/TIA, Sepsis, deep vein thrombosis/pulmonary embolism, AF, ventricular tachycardia, VF
McHugh et al. (2013)	USA	Retrospective cohort	AMI. Sample: 62394 Age: Mdn = 78.0 Sex (%Female): 49.0	NA	30‐day readmission. Risk adjustment	Linked data. To determine the relationship between hospital nursing; i.e. nurse work environment, nurse staffing levels, and nurse education, and 30‐day readmissions among Medicare patients with heart failure, acute myocardial infarction, and pneumonia.	HF, coronary atherosclerosis, AMI, cardiac dysrhythmias, nonspecific chest pain, pneumonia, renal failure, respiratory failure, gastrointestinal haemorrhage	
McManus et al. (2016)	USA	Prospective cohort	ACS. Sample: 2187 Age: µ = 73.0 Sex (%Female): 38.0	NA	Our primary study outcome was whether the patient had an unscheduled readmission at any of our 6 participating hospitals for any reason during the following 30 days.	Data were collected from participants in computer‐assisted face‐to‐face interviews or by phone within 72 hours of discharge. The Telephone Interview for Cognitive Status (TICS) was used to assess cognitive status, the Patient Health Questionnaire was used to assess depression, the generalised anxiety disorder questionnaire was used for assessing anxiety. A 4‐item version of the Perceived Stress Scale was used to assess stress. To assess participants’ engagement with health care the Patient Activation Measure (PAM6) was used. To compare the performance of a CMS‐like model to each of 3 models that incorporated a number of variables representing clinical, psychosocial, and socio‐demographic characteristics, respectively	PCI, CABG, CAD/MI, HF, AF, valvular heart disease, TIA/stroke, peripheral vascular disease, diabetes, Chronic kidney disease, dialysis, chronic lung disease, anaemia, Alzheimer's disease, cancer, hypertension, depression, anxiety, stress, cognitively impaired, anterior myocardial infarction, smoking, heavy alcohol consumption	HF, cardiac arrest, cardiogenic shock
Meadows et al. (2012)	USA	Retrospective cohort	ACS with PCI. Sample: 6687 Age: µ = 56.5 Sex (%Female): 22.6	NA	Readmission within 30 days	US administrative claims data. The objective of this study was to characterise the rehospitalisation of patients with acute coronary syndrome receiving percutaneous coronary intervention in the U.S. health benefit plan.	heart failure, stroke, other cardiovascular conditions	
Nuti et al. (2016)	USA	Retrospective cohort	VA and non‐VA AMI. Sample: 140205 Age: VA µ = 75.5, not VA µ = 77.5 **Sex (%Female): 0.0**	NA	30‐day risk‐standardised mortality rates and risk‐standardised readmission rates for VA and non‐VA hospitals. Mean‐aggregated within MSA differences in mortality and readmission rates were also assessed.	CMS standard analytics files and enrolment database as well as VA administrative claims. Objective—To assess and compare mortality and readmission rates among men in VA and non‐VA hospitals. To avoid confounding geographic effects with health care system effects.	VA hospital, prior PCI or CABG or HF or MI or ACS or atherosclerosis or cardiopulmonary respiratory failure/shock, valvular disease, arrythmia, heart disease, hypertension, stroke, cerebrovascular disease, renal failure, COPD, pneumonia, diabetes, dementia, malnutrition, functional disability, peripheral vascular disease, metastatic cancer, psychiatric disorder, chronic liver disease, severe hematologic disorders, iron deficiency, depression, seizure disorder, fibrosis of lung or chronic lung disorder, asthma, end‐stage renal disease, nephritis, urinary tract disorder, UTI, pneumothorax, other lung disorders, fluid/electrolyte disorders, psychiatric disorders, drug/alcohol abuse, peptic ulcer, GI tract disorders, Parkinson's/Huntington's disease, vertebral fractures, sepsis	
Przybysz‐Zdunek et al. (2012)	Poland	Retrospective cohort	PCI with admission related to ICD−9‐CM code. Sample: 2039 Age: µ = 65.7 Sex (%Female): 31.8	NA	Readmission within 30 days	National Health Fund registry. The aim of this study was to assess rehospitalisation and repeat revascularisation within 30 days of the initial hospitalisation for PCI, using data from Opolskie Voivodeship, National Health Fund (NHF) Registry.	diabetes, congestive heart failure, chronic renal insufficiency, hypertension, peripheral artery disease, bradyarrhythmia, atrial fibrillation, cardiac arrest	
Southern et al. (2014)	Canada	Retrospective cohort	ACS. Sample: 3411 Age: µ = 65.6 Sex (%Female): 30.9	NA	Primary outcomes were inpatient and emergency department–only readmissions, at 30 days.	APPROACH database. To profile the timing, main diagnoses and survival outcomes of inpatient and emergency department readmissions after acute coronary syndrome (ACS).	HF, diabetes, cancer, liver disease, renal disease, pulmonary disease, peripheral vascular disease, dementia, cerebrovascular disease, peptic ulcer disease, rheumatic disease, paraplegia, HIV, MI, renal disease, heart failure	
Tripathi et al. (2017)	USA	Retrospective cohort	PCI with inpatient admission related to ICD−9‐CM code. Sample: 206869 Age: µ = 65.0 Sex (%Female): 32.2	NA	30‐day readmission and readmission costs	National readmission database. The objectives of this study were to evaluate the rate of post‐PCI 30‐day readmission and the associated costs in a cohort of patients who had inpatient PCI. In addition, we examined the factors associated with the risk of 30‐day readmissions and higher costs after accounting for all insurance types, geographical variations, and individual‐ and hospital‐level factor	ischaemic heart disease, heart failure, peripheral artery disease, chronic pulmonary disease, diabetes, renal failure	
Tripathi et al. (2019)	USA	Retrospective cohort	PCI in STEMI patients. Sample: 22257 Age: µ = 62.3 Sex (%Female): 26.0	NA	The primary outcome was the 30‐day readmission rate in the cohort and secondary outcomes were factors associated with readmission. Causes of readmission were identified using ICD−9 codes in primary diagnosis filed during readmission observation. We identified 445 different ICD−9‐CM diagnosis codes and combined the ones with similar diagnoses to form clinically important groups.	National readmission database. To explore pattern, causes and factors associated with 30‐day readmission after multivessel PCI in STEMI patients.	obesity, coagulopathy, chronic kidney disease, hypothyroidism, hypertension, diabetes, congestive heart failure, chronic pulmonary disease, peripheral vascular disease, anaemia, neurological disorder including paralysis, rheumatological disorder, psychiatric disorder including drug abuse	
Zabawa et al. (2018)	France	Retrospective cohort	AMI. Sample: 624 Age: Readmitted µ = 79.2; Non‐readmitted µ = 78.1 Sex (%Female): Readmitted patients = 56.9; non‐readmitted patients = 57.1	NA	The primary outcome was the first all‐cause 30‐day rehospitalisation in an acute care hospital, in the same or another hospital.	Linked data. To investigate the association between Post‐discharge ambulatory care and 30‐day rehospitalisation after discharge of elderly patients hospitalised for AMI, after adjusting for these factors.	diabetes, congestive heart failure, acute kidney failure, chronic kidney failure, atrial fibrillation, coronary heart disease	
